# Systematic literature review of machine learning methods used in the analysis of real-world data for patient-provider decision making

**DOI:** 10.1186/s12911-021-01403-2

**Published:** 2021-02-15

**Authors:** Alan Brnabic, Lisa M. Hess

**Affiliations:** 1 Eli Lilly and Company, Sydney, NSW Australia; 2 Eli Lilly and Company, Indianapolis, IN USA

**Keywords:** Machine learning, Decision making, Decision tree, Random forest, Automated neural network

## Abstract

**Background:**

Machine learning is a broad term encompassing a number of methods that allow the investigator to learn from the data. These methods may permit large real-world databases to be more rapidly translated to applications to inform patient-provider decision making.

**Methods:**

This systematic literature review was conducted to identify published observational research of employed machine learning to inform decision making at the patient-provider level. The search strategy was implemented and studies meeting eligibility criteria were evaluated by two independent reviewers. Relevant data related to study design, statistical methods and strengths and limitations were identified; study quality was assessed using a modified version of the Luo checklist.

**Results:**

A total of 34 publications from January 2014 to September 2020 were identified and evaluated for this review. There were diverse methods, statistical packages and approaches used across identified studies. The most common methods included decision tree and random forest approaches. Most studies applied internal validation but only two conducted external validation. Most studies utilized one algorithm, and only eight studies applied multiple machine learning algorithms to the data. Seven items on the Luo checklist failed to be met by more than 50% of published studies.

**Conclusions:**

A wide variety of approaches, algorithms, statistical software, and validation strategies were employed in the application of machine learning methods to inform patient-provider decision making. There is a need to ensure that multiple machine learning approaches are used, the model selection strategy is clearly defined, and both internal and external validation are necessary to be sure that decisions for patient care are being made with the highest quality evidence. Future work should routinely employ ensemble methods incorporating multiple machine learning algorithms.

## Background

Traditional methods of analyzing large real-world databases (big data) and other observational studies are focused on the outcomes that can inform at the population-based level. The findings from real-world studies are relevant to populations as a whole, but the ability to predict or provide meaningful evidence at the patient level is much less well established due to the complexity with which clinical decision making is made and the variety of factors taken into account by the health care provider [1, 2]. Using traditional methods that produce population estimates and measures of variability, it is very challenging to accurately predict how any one patient will perform, even when applying findings from subgroup analyses. The care of patients is nuanced, and multiple non-linear, interconnected factors must be taken into account in decision making. When data are available that are only relevant at the population level, health care decision making is less informed as to the optimal course of care for a given patient.

Clinical prediction models are an approach to utilizing patient-level evidence to help inform healthcare decision makers about patient care. These models are also known as prediction rules or prognostic models and have been used for decades by health care professionals [3]. Traditionally, these models combine patient demographic, clinical and treatment characteristics in the form of a statistical or mathematical model, usually regression, classification or neural networks, but deal with a limited number of predictor variables (usually below 25). The Framingham Heart Study is a classic example of the use of longitudinal data to build a traditional decision-making model. Multiple risk calculators and estimators have been built to predict a patient’s risk of a variety of cardiovascular outcomes, such as atrial fibrillation and coronary heart disease [4–6]. In general, these studies use multivariable regression evaluating risk factors identified in the literature. Based on these findings, a scoring system is derived for each factor to predict the likelihood of an adverse outcome based on a patient’s score across all risk factors evaluated.

With the advent of more complex data collection and readily available data sets for patients in routine clinical care, both sample sizes and potential predictor variables (such as genomic data) can exceed the tens of thousands, thus establishing the need for alternative approaches to rapidly process a large amount of information. Artificial intelligence (AI), particularly machine learning methods (a subset of AI), are increasingly being utilized in clinical research for prediction models, pattern recognition and deep-learning techniques used to combine complex information for example genomic and clinical data [7–9]. In the health care sciences, these methods are applied to replace a human expert to perform tasks that would otherwise take considerable time and expertise, and likely result in potential error. The underlying concept is that a machine will learn by trial and error from the data itself, to make predictions without having a pre-defined set of rules for decision making. Simply, machine learning can simply be better understood as “learning from data.” [8].

There are two types of learning from the data, unsupervised and supervised. Unsupervised learning is a type of machine learning algorithm used to draw inferences from datasets consisting of input data without labelled responses. The most common unsupervised learning method is cluster analysis, which is used for exploratory data analysis to find hidden patterns or grouping in data. Supervised learning involves making a prediction based on a set of pre-specified input and output variables. There are a number of statistical tools used for supervised learning. Some examples include traditional statistical prediction methods like regression models (e.g. regression splines, projection pursuit regression, penalized regression) that involve fitting a model to data, evaluating the fit and estimating parameters that are later used in a predictive equation. Other tools include tree-based methods (e.g. classification and regression trees [CART] and random forests), which successively partition a data set based on the relationships between predictor variables and a target (outcome) variable. Other examples include neural networks, discriminant functions and linear classifiers, support vector classifiers and machines. Often, predictive tools are built using various forms of model aggregation (or ensemble learning) that may combine models based on resampled or re-weighted data sets. These different types of models can be fitted to the same data using model averaging.

Classical statistical regression methods used for prediction modeling are well understood in the statistical sciences and the scientific community that employs them. These methods tend to be transparent and are usually hypothesis driven but can overlook complex associations with limited flexibility when a high number of variables are investigated. In addition, when using classic regression modeling, choosing the ‘right’ model is not straightforward. Non-traditional machine learning algorithms, and machine learning approaches, may overcome some of these limitations of classical regression models in this new era of big data, but are not a complete solution as they must be considered in the context of the limitations of data used in the analysis [2].

While machine learning methods can be used for both population-based models as well as for informed patient-provider decision making, it is important to note that the data, model, and outputs used to inform the care of an individual patient must meet the highest standards of research quality, as the choice made will likely have an impact on both the long- and short-term patient outcomes. While a range of uncertainty can be expected for population-based estimates, the risk of error for patient level models must be minimized to ensure quality patient care. The risks and concerns of utilizing machine learning for individual patient decision making have been raised by ethicists [10]. The risks are not limited to the lack of transparency, limited data regarding the confidence of the findings, and the risk of reducing patient autonomy in choice by relying on data that may foster a more paternalistic model of healthcare. These are all important and valid concerns, and therefore the role of machine learning for patient care must meet the highest standards to ensure that shared, not simply informed, evidence-based decision making be supported by these methods.

A systematic literature review was published in 2018 that evaluated the statistical methods that have been used to enable large, real-world databases to be used at the patient-provider level [11]. Briefly, this study identified a total of 115 articles that evaluated the use of logistic regression (n = 52, 45.2%), Cox regression (n = 24, 20.9%), and linear regression (n = 17, 14.8%). However, an interesting observation noted several studies utilizing novel statistical approaches such as machine learning, recursive partitioning, and development of mathematical algorithms to predict patient outcomes. More recently, publications are emerging describing the use of Individualized Treatment Recommendation algorithms and Outcome Weighted Learning for personalized medicine using large observational databases [12, 13]. Therefore, this systematic literature review was designed to further pursue this observation to more comprehensively evaluate the use of machine learning methods to support patient-provider decision making, and to critically evaluate the strengths and weaknesses of these methods. For the purposes of this work, data supporting patient-provider decision making was defined as that which provided information specifically on a treatment or intervention choice; while both population-based and risk estimator data are certainly valuable for patient care and decision making, this study was designed to evaluate data that would specifically inform a choice for the patient with the provider. The overarching goal is to provide evidence of how large datasets can be used to inform decisions at the patient level using machine learning-based methods, and to evaluate the quality of such work to support informed decision making.

## Methods

This study originated from a systematic literature review that was conducted in MEDLINE and PsychInfo; a refreshed search was conducted in September 2020 to obtain newer publications (Table 1). Eligible studies were those that analyzed prospective or retrospective observational data, reported quantitative results, and described statistical methods specifically applicable to patient-level decision making. Specifically, patient-level decision making referred to studies that provided data for or against a particular intervention at the patient level, so that the data could be used to inform decision making at the patient-provider level. Studies did not meet this criterion if only a population-based estimates, mortality risk predictors, or satisfaction with care were evaluated. Additionally, studies designed to improve diagnostic tools and those evaluating health care system quality indicators did not meet the patient-provider decision-making criterion. Eligible statistical methods for this study were limited to machine learning-based approaches. Eligibility was assessed by two reviewers and any discrepancies were discussed; a third reviewer was available to serve as a tie breaker in case of different opinions. The final set of eligible publications were then abstracted into a Microsoft Excel document. Study quality was evaluated using a modified Luo scale, which was developed specifically as a tool to standardize high-quality publication of machine learning models [14]. A modified version of this tool was utilized for this study; specifically, the optional item were removed, and three terms were clarified: item 6 (define the prediction problem) was redefined as “define the model,” item 7 (prepare data for model building) was renamed “model building and validation,” and item 8 (build the predictive model) was renamed “model selection” to more succinctly state what was being evaluated under each criterion. Data were abstracted and both extracted data and the Luo checklist items were reviewed and verified by a second reviewer to ensure data comprehensiveness and quality. In all cases of differences in eligibility assessment or data entry, the reviewers met and ensured agreement with the final set of data to be included in the database for data synthesis, with a third reviewer utilized as a tie breaker in case of discrepancies. Data were summarized descriptively and qualitatively, based on the following categories: publication and study characteristics; patient characteristics; statistical methodologies used, including statistical software packages; strengths and weaknesses; and interpretation of findings.

**Table 1:** Search strategy

1	(randomized controlled trial or controlled clinical trial).pt. or randomized.ab
2	Prospective Studies/ or observational trials.mp. or observational research.mp
3	Retrospective Studies/ or retrospective observational.mp. or retrospective database.mp
4	Cross-Sectional Studies/ or cross-sectional.mp
5	(systematic adj2 review).mp
6	1 or 2 or 3 or 4 or 5
7	*Neoplasms/
8	*Cardiovascular Diseases/
9	*Diabetes Mellitus, Type 1/ or *Diabetes Mellitus, Type 2/ or *Diabetes Mellitus/
10	*Autoimmune Diseases/
11	*Alzheimer Disease/
12	7 or 8 or 9 or 10 or 11
13	(decision making or decision analysis).mp. or *Decision Making/ or Decision Support Techniques/
14	Physician–Patient Relations/ or Patient-Centered Care/ or patient cent*.mp
15	nomograms.mp. or Nomograms/
16	prediction model*.mp
17	Patient Preference/ or discrete choice.mp. or conjoint analysis.mp
18	Decision Support Techniques/ or (decision adj2 tool).mp. or decision aid.mp
19	13 or 14 or 15 or 16 or 17 or 18
20	6 and 12 and 19
21	limit 20 to (english language and humans and yr = "2000 -Current")
22	limit 20 to (english language and humans and yr = "2014 -Current")
23	"machine learning".mp. or Machine Learning/ or "Neural Networks (Computer)"/ or Computer Simulation/ or Algorithms/
24	22 and 23
25	data mining.mp. or Data Mining/ or Medical Informatics/
26	22 and 25
27	24 or 26
28	neural network*.mp. or LTSM.ab. or LTSM.ti. or memory network*.mp
29	28 and 6 and 19 and 19
30	limit 29 to (english language and humans and yr = "2014 -Current")
31	27 or 30

## Results

**Fig. 1:**
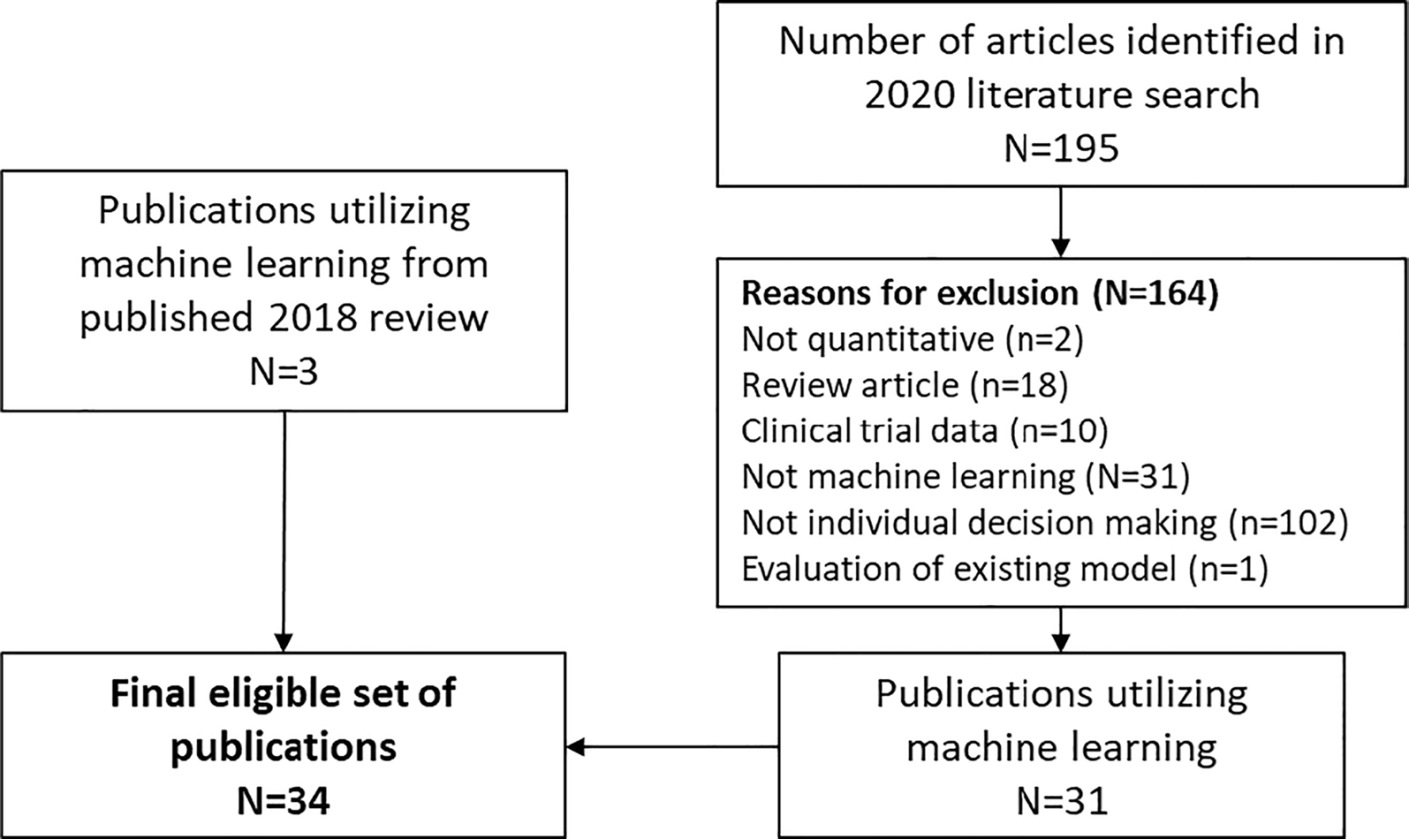
Prisma diagram of screening and study identification

The search strategy was run on September 1, 2020 and identified a total of 34 publications that utilized machine learning methods for individual patient-level decision making (Fig. 1). The most common reason for study exclusion, as expected, was due to the study not meeting the patient-level decision making criterion. A summary of the characteristics of eligible studies and the patient data are included in Table 2. Most of the real-world data sources included retrospective databases or designs (n = 27, 79.4%), primarily utilizing electronic health records. Six analyses utilized prospective cohort studies and one utilized data from a cross sectional study.

**Table 2:** Summary of eligible publications

References	Study objective	Disease state	Data source(s)	Statistical modeling method(s)	Software	Sample size	Number of different models
Alaa et al. [23]	To develop machine-learning-based risk prediction models	Cardiovascular disease	Prospective cohort study—UK Biobank	Cox proportional hazards models, linear support vector machines, random forest, neural networks, AdaBoost, and gradient boosting machines	Python	423,604	6
Anderson et al. [48]	To identify patient characteristics that predict progression to prediabetes and type 2 diabetes in a US adult population	Diabetes	Retrospective database—electronic health records (Humedica)	Novel analytical platform based on a Bayesian approach	Reverse Engineering and Forward Simulation (REFS™)	24,331	1
Azimi et al. [38]	To select patients for surgery or non-surgical options	Neurology	Retrospective database	Logistic regression, Artificial neural network	SPSS for windows (Version 17.0), STATISTICA 10.0 Neural Networks	346	2
Bannister et al. [22]	To determine the utility of genetic programming for the automatic development of clinical prediction models	Cardiovascular disease	Prospective observational cohort	Cox regression models, Tree-based genetic programming	R Package version 3.0.1 and 3.1.2	3873	2
Baxter et al. [24]	To predict the need for surgical intervention in patients with primary open-angle glaucoma	Neurology	Retrospective database—electronic health records (EpicCare)	Logistic regression, random forest, artificial neural network	Random Forest R package; nnet package in R	385	3
Bertsimas et al. [21]	To predict patients at high risk of mortality before the start of treatment regimes	Oncology	Retrospective database—electronic health records and social security death index	Logistic regression, Decision tree analysis (Gradient boosted, optimal classification, and Classification and Regression Tree [CART])	Not specified	23,983	2
Bowman et al. [39]	To develop and validate a comprehensive, multivariate prognostic model for carpal tunnel surgery	Neurology	Retrospective database—clinic data	Logistic regression, artificial neural network	Stata v 14, MATLAB v. 8.3.0.532	200	2
Dong et al. [25]	To present and validate a novel surgical predictive model to facilitate therapeutic decision-making	Inflammatory bowel disease	Retrospective database- electronic health records	Random forest, logistic regression, decision tree, support vector machine, artificial neural network	Python 3.6	239	5
Hearn et al. [40]	To assess whether the prognostication of heart failure patients using cardiopulmonary exercise test data could be improved by considering the entirety of the data generated during a cardiopulmonary exercise test, as opposed to using summary indicators alone	Cardiovascular disease	Retrospective database -electronic health records and exercise test data	Logistic regression, least absolute shrinkage and selection operator (LASSO) model, generalized additive model, feedforward neural network	R Project for Statistical Computing v3.4.2, and Python Programming Language v3.6.2	1156	4
Hertroijs et al. [45]	To identify subgroups of people with newly diagnosed type 2 diabetes with distinct glycaemic trajectories; to predict trajectory membership using patient characteristics	Diabetes	Retrospective database -electronic health records	Latent growth mixture modeling, K-nearest neighbor/Parzen, Fisher, linear/quadratic discriminant classifier, supper vector machine, radial basis function, logistic regression	Mplus Version 7.1	14,305	7
Hill et al. [26]	To develop a clinically applicable risk prediction model to identify associations between baseline and time-varying factors and the identification of atrial fibrillation	Cardiovascular disease	Retrospective database—electronic health records (Clinical Practice Research Datalink, CPRD)	Logistic least absolute shrinkage and selector operator (LASSO), random forests, support vector machines, neural networks	R v3.3.1	2,994,837—baseline model; 162,672—time varying model	4
Hische et al. [20]	To create a simple and reliable tool to identify individuals with impaired glucose metabolism	Diabetes	Cross-sectional study	Decision tree analysis	Quinlan C4.5	1737	1
Isma'eel et al. [41]	To investigate the use of artificial neural networks to improve risk stratification and prediction of myocardial perfusion imaging and angiographic results	Cardiovascular disease	Retrospective medical records	Artificial neural network	Not specified	5354	1
Isma'eel et al. [42]	To compare artificial neural network-based prediction models to other risk models that are being used in clinical practice	Cardiovascular disease	Prospective cohort	Artificial neural network	Not specified	486	1
Jovanovic et al. [43]	to determine whether an artificial neural network model could be constructed to accurately predict the need for therapeutic ERCP in patients with a firm clinical suspicion of having common bile duct stones and to compare it with our previously reported predictive model	Choledocholithiasis	Prospective cohort	Artificial neural network	SPSS v20.0	291	1
Kang et al. [27]	To investigate the feasibility of developing a machine-learning model to predict postinduction hypotension	Cardiovascular disease	Retrospective database—electronic health records	Developed Naïve Bayes, logistic regression, random forest, and artificial neural network	caret R package	222	4
Karhade et al. [28]	To develop algorithms for prediction of prolonged opioid prescription after surgery for lumbar disc herniation	Lumbar disk herniation	Retrospective chart review	Random forest, stochastic gradient boosting, neural network, support vector machine, elastic-net penalized logistic regression	Anaconda Distribution, R version 3.5.0, RStudio version 1.1.453, and Python version 3.6	5413	5
Kebede et al. [29]	to predict CD4 count changes and to identify the predictors of CD4 count changes among patients on ART	HIV/AIDS	Retrospective database and chart review	J48 decision tree/random forest, neural network	WEKA 3.8	3104	2
Khanji et al. [47]	To identify an effective method to build prediction models and assess predictive validity of pre-defined indicators	Cardiovascular disease	Observational trial, cluster randomization	Logistic regression, LASSO regression, Hybrid approach (combination of both approaches)	SAS version 9.1, SPSS version 24, and R version 3.3.2	759	2
Kim et al. [30]	to develop a prediction tool using machine learning for high- or low-risk oncotype dx criteria	Oncology	Retrospective chart review	Two-class Decision Forest, Two-class Decision Jungle, Two-class Bayes Point Machine, Two-class Support Vector Machine, Two-class Neural Network	SAS 9.4; Azure Machine Learning Platform	284	4
Kwon et al. [32]	to predict cardiac arrest using deep learning	Cardiovascular disease	Retrospective database—electronic health records	Random forest, logistic regression, recurrent neural network	Not specified	52,131	3
Kwon et al. [31]	to predict prognosis of out-of-hospital cardiac arrest using deep-learning	Cardiovascular disease	Retrospective database – registry	Logistic regression, support vector machine, random forest	Not specified	36,190	3
Lopez-de-Andres et al. [34]	To estimate predictive factors of in-hospital mortality in patients with type 2 diabetes after major lower extremity amputation	Diabetes	Retrospective database—hospital discharge database	Artificial neural network	Neural Designer; Stata MP version 10.1	40,857 lower extremity amputation events	1
Mubeen et al. [19]	To assess risk of developing Alzheimer’s Disease in mildly cognitively impaired subjects; to classify subjects in two groups: those who would remain stable and those who would progress to develop Alzheimer’s disease	Alzheimer’s Disease	Retrospective database – (Alzheimer’s Disease Neuroimaging Project)	Random Forest algorithm	Random Forest R package	247	1
Neefjes et al. [18]	To develop a prediction model to identify patients with cancer at high risk for delirium	Oncology	Retrospective database –hospital inpatient data	Decision tree analysis	R program Rpart version 3.1; Statistical Package for the Social Sciences (SPSS) v20.0	574	1
Ng et al. [36]	To create a clinical decision support tool to predict survival in cancer patients beyond 120 days after palliative chemotherapy	Oncology	Retrospective database—electronic health records and case notes	Naïve Bayes, neural network, and support vector machine	SIMCA-P + version 12.0.1; SPSS version 19.0; RapidMiner version 5.0.010	325	3
Oviedo et al. [46]	To focus on patient-specific prediction of hypoglycemic events	Diabetes	Retrospective database – hospital clinic data	Support vector classifier	Python	10	1
Pei et al. [17]	To identify individuals with potential diabetes	Diabetes	Retrospective medical records review	Decision tree analysis	WEKA 3.8.1 and SPSS version 20.0	10,436	1
Perez-Gandia et al. [37]	To predict future glucose concentration levels from continuous glucose monitoring data	Diabetes	Retrospective database—device dataset	Artificial neural network	Not specified	15	1
Ramezankhani et al. [16]	To gain more information on interactions between factors contributing to the incidence of type 2 diabetes	Diabetes	Prospective cohort	Decision tree analysis (CART, Quick Unbiased Efficient Statistical Tree [QUEST], and commercial version [C5.0])	IBM SPSS modeler 14.2	6647	1
Rau et al. [35]	To predict the development of liver cancer within 6 years of diagnosis with type 2 diabetes	Diabetes and Oncology	Retrospective database—claims linked to registry data	Logistic regression, artificial neural network, support vector machine, and decision tree analysis	STATISTICA, version 10	2060	4
Scheer et al. [33]	To develop a model based on baseline demographic, radiographic, and surgical factors that can predict if patients will sustain an intraoperative or perioperative major complication	Spinal deformity	Retrospective database	Decision tree analysis	SPSS version 22; SPSS modeler version 16	557	1
Toussi et al. [15]	To identify knowledge gaps in guidelines and to explore physicians' therapeutic decisions using data mining techniques to fill these knowledge gaps	Diabetes	Retrospective database—electronic health records	Decision tree analysis	Quinlan’s C5.0 decision-tree learning algorithm; SPSS Clementine software version 10.1	463	1
Zhou et al. [44]	To assess pre-procedural independent risk factors and to establish a “Risk Prediction for Early Biliary Infection” nomogram for patients with malignant biliary obstruction who underwent percutaneous transhepatic biliary stent	Oncology	Retrospective medical record and trial data	Logistic regression, artificial neural network	SPSS version 22; R package (version 3.4.3)	243	2

### General approaches to machine learning

The types of classification or prediction machine learning algorithms are reported in Table 2. These included decision tree/random forest analyses (19 studies) [15–33] and neural networks (19 studies) [24–30, 32, 34–44]. Other approaches included latent growth mixture modeling [45], support vector machine classifiers [46], LASSO regression [47], boosting methods [23], and a novel Bayesian approach [26, 40, 48]. Within the analytical approaches to support machine learning, a variety of methods were used to evaluate model fit, such as Akaike Information Criterion, Bayesian Information Criterion, and the Lo-Mendel-Rubin likelihood ratio test [22, 45, 47], and while most studies included the area under the curve (AUC) of receiver-operator characteristic (ROC) curves (Table 3), analyses also included sensitivity/specificity [16, 19, 24, 30, 41–43], positive predictive value [21, 26, 32, 38, 40–43], and a variety of less common approaches such as the geometric mean [16], use of the Matthews correlation coefficient (ranges from -1.0, completely erroneous information, to + 1.0, perfect prediction) [46], defining true/false negatives/positives by means of a confusion matrix [17], calculating the root mean square error of the predicted versus original outcome profiles [37], or identifying the model with the best average performance training and performance cross validation [36].

**Table 3:** Details of methods applied to the analysis in eligible studies

References	Internal validation	Evaluation of model fit/performance	Handling of missing values
Alaa et al. [23]	Training set corresponding to 90% of the sample; ten-fold cross validation	Area under the receiver operating characteristic curve and 95% confidence intervals (Wilson score intervals), Brier score	Retained as an informative variable, and only variables missing for 85% or more of participants were excluded
Anderson et al. [48]	Split the data based on site of care	Bayesian Information Criterion, prediction model ensembles, ß estimates, predicted probabilities, and area under the receiver operating characteristic curve estimates	Missing covariate values were included in models as a discrete category
Azimi et al. [38]	2:1:1 ratio to generate training, testing, and validation cohorts	Receiver-operating characteristic curves, positive predictive value, negative predictive value, area under the curve from the receiver operating curve analysis, Homer-Lemeshow statistic	Cases with missing outcome data were excluded
Bannister et al. [22]	Derivation set of approximately 66.67% and a validation set of approximately 33.33% as noted in text (abstract states 70:30 split)	Akaike Information Criterion	Single imputation, followed by multiple imputation in the final model to evaluate differences in model parameters
Baxter et al. [24]	Leave-one-out cross-validation (LOOCV) approach, also known as the jackknife method	Area under the receiver operating characteristic curve, sensitivity, specificity, accuracy, the Youden index	Not mentioned
Bowman [39]	Re-analysis of models using clinic data during a different time period	Area under the receiver operating characteristic curve	Not mentioned
Bertsimas et al. [21]	60/40 split	Positive predictive value, area under the curve, accuracy	Imputed using an optimal-impute algorithm
Dong et al. [25]	9/1 random split, ten-fold cross validation	Accuracy, precision, F1 score, true negative rate, area under the receiver operating characteristic curve	Missing values were filled based on the mean value of data from the same attribute of the same patient. If a patient had fewer than 3 records, imputed the mean value of that attribute from all patients
Hearn et al. [40]	100-iteration Monte Carlo cross validation, 75/25 split, five-fold cross validation	Mean area under the receiver operating characteristic curve, true- and false-positive rates, true- and false-negative rates, positive and negative predictive values	Variables with > 10% of values missing were discarded from the analysis, whereas the remaining missing values were filled in using multiple imputation by chained random forests (maximum number of iterations = 5, number of trees = 10). The sole exception to the 10% cutoff was heart rate recovery, which featured 32% missing values but was kept in the data set and imputed with the above procedure because of its wide usage in prognostication from cardiopulmonary exercise test
Hertroijs et al. [45]	Five-fold cross validation	Akaike Information Criterion, Bayesian Information Criterion, Lo-Mendel-Rubin likelihood ratio test	The full information maximum likelihood method was used for estimating model parameters in the presence of missing data for the development of the model, but patients with missing covariate values at baseline were excluded from the validation of the model
Hill et al. [26]	2/1 split	Area under the receiver operating characteristic curve, positive predictive value, potential number reeded-to-screen	Imputed with last-observation-carried-forward
Hische et al. [20]	Ten-fold cross validation	Models with a mean sensitivity above 90% after cross-validation were selected, specificity, positive predictive value, negative predictive value	Not mentioned
Isma'eel et al. [41]	For myocardial perfusion imaging: 120 out the 479 patients who tested positive were added randomly to the derivation cohort; 120 of the remaining 4875 patients who tested negative were also added randomly to the derivation cohort. The remaining 5114 patients were all added to the validation cohort. For coronary artery disease: the derivation cohort was randomly chosen as follows: 93 out of the 278 patients who tested positive were added randomly to the derivation cohort and 93 out of the remaining 5076 patients who tested negative were also added randomly to the derivation cohort. The remaining 5169 patients are all added to the validation cohort	Sensitivity and specificity, discriminatory power and 95% confidence interval, number of tests avoided, negative predictive value, positive predictive value	Not mentioned
Isma'eel et al. [42]	The derivation cohort was randomly chosen 30 out of the 59 patients who tested positive were added randomly to the derivation cohort, and 30 out of the remaining 427 patients who tested negative were also added randomly to the derivation cohort. The remaining 426 patients (29 positive, 397 negative) were all added to the testing cohort; during the training phase, the 60 patients that are used for training were split 80% for pure training and 20% for validation	Negative and positive predictive values, descriminatory power, percentage of avoided tests, sensitivity and specificity	Not mentioned
Jovanovic et al. [43]	The sample was randomly divided into 3 parts: training, testing, and validation sample	Area under the receiver operating curve, sensitivity, specificity, and positive and negative predictive values	Not mentioned
Kang et al. [27]	Four-fold cross validation, 75/25 split (training/validation)	Area under the receiver operating curve, accuracy, precision, recall	Not mentioned
Karhade et al. [28]	tenfold cross validation	Discrimination (c-statistic or area under the receiver operating curve), calibration (calibration slope, calibration intercept), and overall performance (Brier score)	Multiple imputation with the missForest methodology was undertaken for variables with less than 30% missing data
Kebede et al. [29]	10% cross validation, 90/10 split (training/testing)	Area under the receiver operating curve; classification accuracy-true positive, false positive, precision, recall	If information is incomplete, un-readable or their manual record is lost, patients were excluded from the study
Khanji et al. [47]	Ten-fold cross validation	Akaike Information Criterion, area under the receiver operating curve	Excluded patients with missing data at the end of the study (± 6 months)
Kim et al. [30]	70/30 split (training/testing)	Area under the receiver operating curve, sensitivity, specificity, precision, accuracy	Not mentioned
Kwon et al. [32]	Derivation set (June 2010-July 2016) and validation set (Aug 2016–2017) split by date	Receiver operating characteristic curve, the area under the precision–recall curve, net reclassification index, sensitivity, positive predictive value, negative predictive value, net reclassification index, F-measure	Not mentioned
Kwon et al. [31]	Split into derivation and validation datasets according to the year. The derivation data was the patient data for 2012–2015, and the validation data was the patient data for 2016	Area under the receiver operating characteristic curve	Excluded patients with missing values
Lopez-de-Andres et al. [34]	Random 60/20/20 split, where the third group was selected for model selection purposes prior to validation	Area under the receiver operating characteristic curve, accuracy rate, error rate, sensitivity, specificity, precision, positive likelihood, negative likelihood, F1 score, false positive rate, false negative rate, false discovery rate, positive predictive value, negative predictive value, Matthews correlation, informedness, markedness	Not mentioned
Mubeen et al. [19]	Ten-fold cross validation	Accuracy, sensitivity, specificity based on prediction of out-of-bag samples, area under the receiver operating characteristic curve	Not mentioned
Neefjes et al. [18]	Five-fold cross validation	Area under the receiver operating characteristic curve	Not mentioned
Ng et al. [36]	60/40 split, five-fold cross validation	Area under the curve, sensitivity, specificity, accuracy	Excluded patients with missing data
Oviedo et al. [46]	80/20 split (training/testing)	Matthews correlation coefficient	Removed records from the model with missing data
Pei et al. [17]	70/30 split (training/testing)	True positives, true negatives, false negatives, false positives, area under the receiver operating characteristic curve	Removed patients from the model with missing data
Perez-Gandia et al. [37]	Three subjects (each with two daily profiles) were used for training, the remaining patients were used for validation	Accuracy	Spline techniques were used to impute missing data in the training set
Ramezankhani et al. [16]	70/30 split (training/validation)	Sensitivity, specificity, positive predictive value, negative predictive value, geometric mean, F-measure, area under the curve	Single imputation was used, for continuous variables- CART, for categorical variables- weighted K-Nearest Neighbor approach
Rau et al. [35]	70/30 split, ten-fold cross validation	Sensitivity, specificity, area under the curve	Not mentioned
Scheer et al. [33]	70/30 split (training/testing)	Accuracy, area under the receiver operating characteristic curve, predictor importance	Missing values within the database were imputed using standard techniques such as mean and median imputation
Toussi et al. [15]	Ten-fold cross validation	Precision (proportion of true positive records to the proportion of true positive and false positive records)	imputed using a model-based approach
Zhou et al. [44]	Training cohort: 142 patients were part of a prior phase III randomized controlled trial (Oct 2013–Mar 2016) + 182 eligible consecutive patients (Jan 2012–Dec 2016). Validation cohort: 61 eligible consecutive patients (Jan 2017–Aug 2017)	The concordance index (c-index) was calculated as the area under the receiver operating characteristic curve, calibration plot	Patients were excluded if clinical data were missing before or 30 days after percutaneous transhepatic biliary drainage

### Statistical software packages

The statistical programs used to perform machine learning varied widely across these studies, no consistencies were observed (Table 2). As noted above, one study using decision tree analysis used Quinlan’s C5.0 decision tree algorithm [15] while a second used an earlier version of this program (C4.5) [20]. Other decision tree analyses utilized various versions of R [18, 19, 22, 24, 27, 47], International Business Machines (IBM) Statistical Package for the Social Sciences (SPSS) [16, 17, 33, 47], the Azure Machine Learning Platform [30], or programmed the model using Python [23, 25, 46]. Artificial neural network analyses used Neural Designer [34] or Statistica V10 [35]. Six studies did not report the software used for analysis [21, 31, 32, 37, 41, 42].

### Families of machine learning algorithms

Also as summarized in Table 2, more than one third of all publications (n = 13, 38.2%) applied only one family of machine learning algorithm to model development [16–20, 34, 37, 41–43, 46, 48]; and only four studies utilized five or more methods [23, 25, 28, 45]. One applied an ensemble of six different algorithms and the software was set to run 200 iterations [23], and another ran seven algorithms [45].

### Internal and external validation

**Fig. 2:**
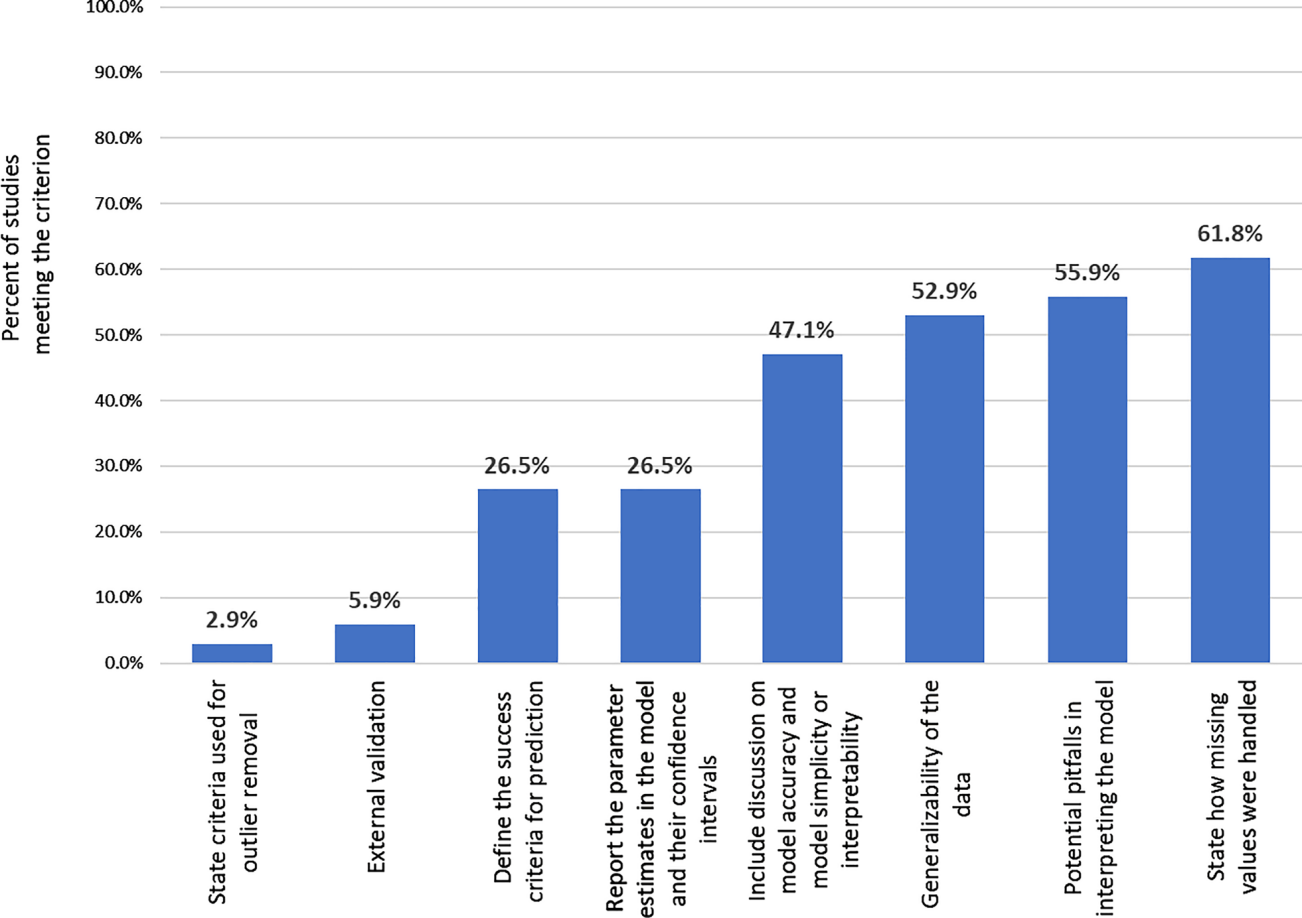
Least frequently met study quality items, modified Luo Scale [14]

Evaluation of study publication quality identified the most common gap in publications as the lack of external validation, which was conducted by only two studies [15, 20]. Seven studies predefined the success criteria for model performance [20, 21, 23, 35, 36, 46, 47], and five studies discussed the generalizability of the model [20, 23, 34, 45, 48]. Six studies [17, 18, 21, 22, 35, 36] discussed the balance between model accuracy and model simplicity or interpretability, which was also a criterion of quality publication in the Luo scale [14]. The items on the checklist that were least frequently met are presented in Fig. 2. The complete quality assessment evaluation for each item in the checklist is included in Additional file 1: Table S1.

There were a variety of approaches taken to validate the models developed (Table 3). Internal validation with splitting into a testing and validation dataset was performed in all studies. The cohort splitting approach was conducted in multiple ways, using a 2:1 split [26], 60/40 split [21, 36], a 70/30 split [16, 17, 22, 30, 33, 35], 75/25 split [27, 40], 80/20 split [46], 90/10 split [25, 29], splitting the data based on site of care [48], a 2/1/1 split for training, testing and validation [38], and splitting 60/20/20, where the third group was selected for model selection purposes prior to validation [34]. Nine studies did not specifically mention the form of splitting approach used [15, 18–20, 24, 29, 39, 45, 47], but most of those noted the use of *k* fold cross validation. One training set corresponded to 90% of the sample [23], whereas a second study was less clear, as input data were at the observation level with multiple observations per patient, and 3 of the 15 patients were included in the training set [37]. The remaining studies did not specifically state splitting the data into testing and validation samples, but most specified they performed five-fold cross validation (including one that generally mentioned cohort splitting) [18, 45] or ten-fold cross validation strategies [15, 19, 20, 28].

External validation was conducted by only two studies (5.9%). Hische and colleagues conducted a decision tree analysis, which was designed to identify patients with impaired fasting glucose [20]. Their model was developed in a cohort study of patients from the Berlin Potsdam Cohort Study (n = 1527) and was found to have a positive predictive value of 56.2% and a negative predictive value of 89.1%. The model was then tested on an independent from the Dresden Cohort (n = 1998) with a family history of type II diabetes. In external validation, positive predictive value was 43.9% and negative predictive value was 90.4% [20]. Toussi and colleagues conducted both internal and external validation in their decision tree analysis to evaluate individual physician prescribing behaviors using a database of 463 patient electronic medical records [15]. For the internal validation step, the cross-validation option was used from Quinlan’s C5.0 decision tree learning algorithm as their study sample was too small to split into a testing and validation sample, and external validation was conducted by comparing outcomes to published treatment guidelines. Unfortunately, they found little concordance between physician behavior and guidelines potentially due to the timing of the data not matching the time period in which guidelines were implemented, emphasizing the need for a contemporaneous external control [15].

### Handling of missing values

Missing values were addressed in most studies (n = 21, 61.8%) in this review, but there were thirteen remaining studies that did not mention if there were missing data or how they were handled (Table 3). For those that reported methods related to missing data, there were a wide variety of approaches used in real-world datasets. The full information maximum likelihood method was used for estimating model parameters in the presence of missing data for the development of the model by Hertroijs and colleagues, but patients with missing covariate values at baseline were excluded from the validation of the model [45]. Missing covariate values were included in models as a discrete category [48]. Four studies removed patients from the model with missing data [46], resulting in the loss of 16%-41% of samples in three studies [17, 36, 47]. Missing data from primary outcome variables were reported among with 59% (men) and 70% (women) within a study of diabetes [16]. In this study, single imputation was used; for continuous variables CART (IBM SPSS modeler V14.2.03) and for categorical variables the authors used the weighted K-Nearest Neighbor approach using RapidMiner (V.5) [16]. Other studies reported exclusion but not specifically the impact on sample size [29, 31, 38, 44]. Imputation was conducted in a variety of ways for studies with missing data [22, 25, 28, 33]. Single imputation was used in the study by Bannister and colleagues, but followed by multiple imputation in the final model to evaluate differences in model parameters [22]. One study imputed with a standard last-imputation-forward approach [26]. Spline techniques were used to impute missing data in the training set of one study [37]. Missingness was largely retained as an informative variable, and only variables missing for 85% or more of participants were excluded by Alaa et al. [23] while Hearn et al. used a combination of imputation and exclusion strategies [40]. Lastly, missing or incomplete data were imputed using a model-based approach by Toussi et al. [15] and using an optimal-impute algorithm by Bertsimas et al. [21].

### Strengths and weaknesses noted by authors

Publications summarized the strengths and weaknesses of the machine learning methods employed. Low complexity and simplicity of machine-based learning models were noted as strengths of this approach [15, 20]. Machine learning approaches were both powerful and efficient methods to apply to large datasets [19]. It was noted that parameters in this study that were significant at the patient level were included, even if at the broader population-based level using traditional regression analysis model development they would have not been significant and therefore would have been otherwise excluded using traditional approaches [34]. One publication noted the value of machine learning being highly dependent on the model selection strategy and parameter optimization, and that machine learning in and of itself will not provide better estimates unless these steps are conducted properly [23].

Even when properly planned, machine learning approaches are not without issues that deserve attention in future studies that employ these techniques. Within the eligible publications, weaknesses included overfitting the model with the inclusion of too much detail [15]. Additional limitations are based on the data sources used for machine learning, such as the lack of availability of all desired variables and missing data that can affect the development and performance of these models [16, 34, 36, 48]. The lack of all relevant variables was noted as a particular concern for retrospective database studies, where the investigator is limited to what has been recorded [26, 28, 29, 38, 40]. Importantly and as observed in the studies included in this review, the lack of external validation was stated as a limitation of studies included in this review [28, 30, 38, 42].

Limitations can also be on the part of the research team, as the need for both clinical and statistical expertise in the development and execution of studies using machine learning-based methodology, and users are warned against applying these methods blindly [22]. The importance of the role of clinical and statistical experts in the research team was noted in one study and highlighted as a strength of their work [21].

## Discussion

This study systematically reviewed and summarized the methods and approaches used for machine learning as applied to observational datasets that can inform patient-provider decision making. Machine learning methods have been applied much more broadly across observational studies than in the context of individual decision making, so the summary of this work does not necessarily apply to all machine learning-based studies. The focus of this work is on an area that remains largely unexplored, which is how to use large datasets in a manner that can inform and improve patient care in a way that supports shared decision making with reliable evidence that is applicable to the individual patient. Multiple publications cite the limitations of using population-based estimates for individual decisions [49–51]. Specifically, a summary statistic at the population level does not apply to each person in that cohort. Population estimates represent a point on a potentially wide distribution, and any one patient could fall anywhere within that distribution and be far from the point estimate value. On the other extreme, case reports or case series provide very specific individual-level data, but are not generalizable to other patients [52]. This review and summary provides guidance and suggestions of best practices to improve and hopefully increase the use of these methods to provide data and models to inform patient-provider decision making.

It was common for single modeling strategies to be employed within the identified publications. It has long been known that single algorithms to estimation can produce a fair amount of uncertainty and variability [53]. To overcome this limitation, there is a need for multiple algorithms and multiple iterations of the models to be performed. This, combined with more powerful analytics in recent years, provides a new standard for machine learning algorithm choice and development. While in some cases, a single model may fit the data well and provide an accurate answer, the certainty of the model can be supported through novel approaches, such as model averaging [54]. Few studies in this review combined multiple families of modeling strategies along with multiple iterations of the models. This should become a best practice in the future and is recommended as an additional criterion to assess study quality among machine learning-based modeling [54].

External validation is critical to ensure model accuracy, but was rarely conducted in the publications included in this review. The reasons for this could be many, such as lack of appropriate datasets or due to the lack of awareness of the importance of external validation [55]. As model development using machine learning increases, there is a need for external validation prior to application of models in any patient-provider setting. The generalizability of models is largely unknown without these data. Publications that did not conduct external validation also did not note the need for this to be completed, as generalizability was discussed in only five studies, one of which had also conducted the external validation. Of the remaining four studies, the role of generalizability was noted in terms of the need for future external validation in only one study [48]. Other reviews that were more broadly conducted to evaluate machine learning methods similarly found a low rate of external validation (6.6% versus 5.9% in this study) [56]. It was shown that there was lower prediction accuracy by external validation than simply by cross validation alone. The current review, with a focus on machine learning to support decision making at a practical level, suggests external validation is an important gap that should be filled prior to using these models for patient-provider decision making.

Luo and others suggest that *k*-fold validation may be used with proper stratification of the response variable as part of the model selection strategy [14, 55]. The studies identified in this review generally conducted 5- or tenfold validation. There is no formal rule for the selection for the value of *k*, which is typically based on the size of the dataset; as *k* increases, bias will be reduced, but in turn variance will increase. While the tradeoff has to be accounted for, *k* = 5–10 has been found to be reasonable for most study purposes [57].

The evidence from identified publications suggests that the ethical concerns of lack of transparency and failure to report confidence in the findings are largely warranted. These limitations can be addressed through the use of multiple modeling approaches (to clarify the ‘black box’ nature of these approaches) and by including both external and high k-fold validation (to demonstrate the confidence in findings). To ensure these methods are used in a manner that improves patient care, the expectations of population-based risk prediction models of the past are no longer sufficient. It is essential that the right data, the right set of models, and appropriate validation are employed to ensure that the resulting data meet standards for high quality patient care.

This study did not evaluate the quality of the underlying real-world data used to develop, test or validate the algorithms. While not directly part of the evaluation in this review, researchers should be aware that all limitations of real-world data sources apply regardless of the methodology employed. However, when observational datasets are used for machine learning-based research, the investigator should be aware of the extent to which the methods they are using depend on the data structure and availability, and should evaluate a proposed data source to ensure it is appropriate for the machine learning project [45]. Importantly, databases should be evaluated to fully understand the variables included, as well as those variables that may have prognostic or predictive value, but may not be included in the dataset. The lack of important variables remains a concern with the use of retrospective databases for machine learning. The concerns with confounding (particularly unmeasured confounding), bias (including immortal time bias), and patient selection criteria to be in the database must also be evaluated [58, 59]. These are factors that should be considered prior to implementing these methods, and not always at the forefront of consideration when applying machine learning approaches. The Luo checklist is a valuable tool to ensure that any machine-learning study meets high research standards for patient care, and importantly includes the evaluation of missing or potentially incorrect data (i.e. outliers) and generalizability [14]. This should be supplemented by a thorough evaluation of the potential data to inform the modeling work prior to its implementation, and ensuring that multiple modeling methods are applied.

## Conclusions

This review found a wide variety of approaches, methods, statistical software and validation strategies that were employed in the application of machine learning methods to inform patient-provider decision making. Based on these findings, there is a need to ensure that multiple modeling approaches are employed in the development of machine learning-based models for patient care, which requires the highest research standards to reliably support shared evidence-based decision making. Models should be evaluated with clear criteria for model selection, and both internal and external validation are needed prior to applying these models to inform patient care. Few studies have yet to reach that bar of evidence to inform patient-provider decision making.

## Supplementary Information

**Additional file 1.** **Table S1.** Study quality of eligible publications, modified Luo scale [14].

## Data Availability

All data generated or analyzed during this study are included in this published article and its supplementary information files.

## Abbreviations

AI: Artificial intelligence
AUC: Area under the curve
CART: Classification and regression trees
LASSO: Logistic least absolute shrinkage and selector operator
