# Supplementary material for: Systematic literature review of machine learning methods used in the analysis of real-world data for patient-provider decision making
Source: BMC Med Inform Decis Mak. 2021 Feb 15;21:54. doi: 10.1186/s12911-021-01403-2 (PMC7885605; doi:10.1186/s12911-021-01403-2)
Supplement: Supplementary file 1 — Additional file 1. Table S1. Study quality of eligible publications, modified Luo scale [14]. [file 12911_2021_1403_MOESM1_ESM.docx]

**Supplemental table 1.** Study quality of eligible publications, modified Luo scale (14)

| **Section** | **Checklist item** | Alaa 2019 (23) | Anderson 2016 (48) | Azimi 2017 (38) | Bannister 2018 (22) | Baxter 2019 (24) | Bertsimas 2018 (21) | Bowman 2018 (39) | Dong 2018 (25) | Hearn 2019 (40) | Hertroijs 2018 (45) | Hill 2019 (26) | Hische 2010 (20) |
| --- | --- | --- | --- | --- | --- | --- | --- | --- | --- | --- | --- | --- | --- |
| Title | Identify the report as introducing a model | Yes | Yes | Yes | Yes | Yes | No | Yes | Yes | Yes | No | Yes | Yes |
| Abstract | Background | Yes | Yes | Yes | No | No | Yes | Yes | Yes | Yes | No | Yes | Yes |
|  | Objectives | Yes | Yes | Yes | Yes | Yes | Yes | No | Yes | Yes | Yes | Yes | Yes |
|  | Data sources | Yes | Yes | No | Yes | Yes | Yes | No | Yes | Yes | Yes | Yes | No |
|  | Machine learning methods applied | Yes | Yes | Yes | Yes | Yes | Yes | Yes | Yes | Yes | Yes | Yes | Yes |
|  | Key result(s) | Yes | Yes | Yes | Yes | Yes | Yes | Yes | Yes | Yes | Yes | Yes | Yes |
|  | Conclusion including the practical value of the model | Yes | Yes | Yes | No | Yes | Yes | Yes | Yes | Yes | Yes | Yes | Yes |
| Intro-duction | Identify the clinical goal | Yes | Yes | Yes | No | Yes | Yes | Yes | Yes | Yes | Yes | Yes | Yes |
|  | Review the current practice and existing models | Yes | Yes | Yes | Yes | Yes | Yes | No | Yes | Yes | No | Yes | Yes |
|  | State the nature of study as being a model based on machine learning methods | Yes | Yes | Yes | Yes | Yes | Yes | No | Yes | No | No | Yes | Yes |
|  | Identify how the study may benefit the clinical goal | Yes | Yes | Yes | No | Yes | Yes | No | Yes | Yes | Yes | Yes | Yes |
| Methods | Identify the clinical setting for the target model. | Yes | Yes | Yes | Yes | Yes | Yes | Yes | Yes | Yes | Yes | Yes | Yes |
|  | Specify if the study is retrospective or prospective. | Yes | Yes | Yes | Yes | Yes | Yes | Yes | Yes | Yes | Yes | Yes | No |
|  | Specify if the model is prognostic or diagnostic | Yes | Yes | Yes | Yes | Yes | Yes | Yes | Yes | Yes | Yes | Yes | Yes |
|  | Identify relevant data sources and quote the ethics approval for data access. | Yes | Yes | Yes | No | Yes | Yes | Yes | Yes | Yes | Yes | Yes | Yes |
|  | State the inclusion and exclusion criteria for data. | Yes | Yes | Yes | Yes | Yes | Yes | Yes | Yes | Yes | Yes | Yes | Yes |
|  | Describe the time span of data and the sample or cohort size. | Yes | Yes | Yes | Yes | Yes | Yes | Yes | Yes | Yes | Yes | Yes | No |
|  | Define the variables | Yes | Yes | Yes | Yes | Yes | Yes | Yes | Yes | Yes | Yes | Yes | Yes |
|  | Describe the basic statistics of the dataset, particularly of the response variable. | Yes | Yes | Yes | Yes | Yes | Yes | Yes | Yes | Yes | Yes | Yes | Yes |
|  | Define how outcomes are measured | Yes | Yes | Yes | Yes | Yes | Yes | Yes | Yes | Yes | Yes | Yes | Yes |
|  | Determine the form of the model (e.g., classification, regression). | Yes | Yes | Yes | Yes | Yes | Yes | Yes | Yes | Yes | Yes | Yes | Yes |
|  | Define the success criteria for prediction | Yes | No | No | No | No | Yes | Yes | No | No | No | No | Yes |
|  | Specify the modeling techniques. If only one type of model was used, justify the decision for using that model. | Yes | Yes | Yes | Yes | Yes | Yes | Yes | Yes | Yes | Yes | Yes | Yes |
|  | State if outliers with impossible or extreme responses are removed; state any criteria used for outlier removal. | No | No | No | No | No | No | No | No | No | Yes | No | No |
|  | State how missing values were handled. | Yes | Yes | Yes | Yes | No | Yes | No | Yes | Yes | Yes | Yes | No |
|  | Split the data into a derivation/training set and a validation/ test set. | Yes | Yes | Yes | Yes | Yes | Yes | Yes | Yes | Yes | Yes | Yes | Yes |
|  | Specify the model validation strategies | Yes | Yes | Yes | Yes | Yes | Yes | Yes | Yes | Yes | Yes | Yes | Yes |
|  | Implement the model selection strategy | Yes | No | No | No | Yes | No | Yes | Yes | Yes | Yes | Yes | Yes |
|  | External validation should also be performed whenever possible. | No | No | No | No | No | No | No | No | No | No | No | Yes |
|  | Report the validation metrics. | Yes | Yes | Yes | Yes | Yes | Yes | Yes | Yes | Yes | Yes | Yes | Yes |
| Results | Assess whether sufficient data were available for a good fit of the model | Yes | Yes | Yes | Yes | Yes | Yes | Yes | Yes | Yes | Yes | Yes | Yes |
|  | Report the predictive performance of the final model in terms of the validation metrics specified in the methods section. | Yes | Yes | Yes | Yes | Yes | Yes | Yes | Yes | Yes | Yes | Yes | Yes |
|  | If possible, report the parameter estimates in the model and their confidence intervals or report non-parametric estimates from bootstrap samples. | No | Yes | No | Yes | Yes | No | No | No | Yes | No | No | No |
|  | If possible, report what variables were shown to be predictive/prognostic of the outcome variable. | Yes | Yes | No | Yes | Yes | Yes | Yes | Yes | Yes | Yes | Yes | Yes |
|  | Report findings from internal/ external validation | Yes | Yes | Yes | Yes | Yes | No | Yes | Yes | Yes | Yes | Yes | Yes |
| Discus-sion | Interpretation of the final model. | Yes | Yes | Yes | Yes | Yes | Yes | Yes | Yes | Yes | Yes | Yes | Yes |
|  | Report the clinical implications derived from the obtained predictive performance. | Yes | Yes | No | Yes | Yes | Yes | Yes | Yes | Yes | Yes | Yes | Yes |
|  | Include discussion on (1) balance between model accuracy and model simplicity or interpretability, and (2) the familiarity with the modeling techniques of the end user. | No | No | No | Yes | No | Yes | Yes | No | Yes | No | Yes | No |
|  | Discuss model in context of other models in the literature | Yes | Yes | No | No | Yes | Yes | Yes | Yes | Yes | Yes | Yes | Yes |
|  | Potential pitfalls in interpreting the model | Yes | No | No | Yes | No | No | Yes | Yes | No | No | Yes | Yes |
|  | Potential bias of the data used in modeling | Yes | Yes | Yes | No | Yes | Yes | Yes | No | Yes | No | Yes | Yes |
|  | Generalizability of the data | Yes | Yes | Yes | No | No | No | Yes | Yes | No | Yes | Yes | Yes |

| **Section** | **Checklist item** | Isma’eel 2016 (41) | Isma’eel 2018 (42) | Jovanovic 2014 (43) | Kang 2020 (27) | Karhade 2019 (28) | Kebede 2017 (29) | Khanji 2019 (47) | Kim 2019 (30) | Kwon 2018 (32) | Kwon 2019 (31) | Lopez-de-Andres 2016 (34) |
| --- | --- | --- | --- | --- | --- | --- | --- | --- | --- | --- | --- | --- |
| Title | Identify the report as introducing a model | Yes | Yes | Yes | Yes | Yes | Yes | Yes | Yes | Yes | Yes | Yes |
| Abstract | Background | Yes | Yes | Yes | Yes | Yes | Yes | Yes | Yes | Yes | Yes | Yes |
|  | Objectives | Yes | Yes | Yes | Yes | Yes | Yes | Yes | Yes | Yes | Yes | Yes |
|  | Data sources | Yes | Yes | Yes | Yes | Yes | Yes | Yes | Yes | Yes | Yes | Yes |
|  | Machine learning methods applied | Yes | Yes | Yes | Yes | Yes | Yes | Yes | Yes | Yes | Yes | Yes |
|  | Key result(s) | Yes | Yes | Yes | Yes | Yes | Yes | Yes | Yes | Yes | Yes | Yes |
|  | Conclusion including the practical value of the model | Yes | Yes | Yes | Yes | Yes | Yes | Yes | Yes | Yes | Yes | Yes |
| Intro-duction | Identify the clinical goal | Yes | Yes | Yes | Yes | Yes | Yes | Yes | Yes | Yes | Yes | Yes |
|  | Review the current practice and existing models | Yes | No | No | Yes | Yes | Yes | Yes | Yes | Yes | Yes | Yes |
|  | State the nature of study as being a model based on machine learning methods | Yes | Yes | Yes | Yes | No | Yes | Yes | Yes | Yes | Yes | Yes |
|  | Identify how the study may benefit the clinical goal | Yes | Yes | Yes | Yes | Yes | Yes | Yes | Yes | Yes | Yes | Yes |
| Methods | Identify the clinical setting for the target model. | Yes | Yes | Yes | Yes | Yes | Yes | Yes | Yes | Yes | Yes | Yes |
|  | Specify if the study is retrospective or prospective. | Yes | Yes | Yes | Yes | Yes | No | Yes | Yes | Yes | Yes | Yes |
|  | Specify if the model is prognostic or diagnostic | Yes | Yes | Yes | Yes | Yes | Yes | Yes | Yes | Yes | Yes | Yes |
|  | Identify relevant data sources and quote the ethics approval for data access. | Yes | Yes | Yes | Yes | Yes | Yes | Yes | Yes | Yes | Yes | Yes |
|  | State the inclusion and exclusion criteria for data. | Yes | Yes | Yes | Yes | Yes | Yes | Yes | Yes | Yes | Yes | Yes |
|  | Describe the time span of data and the sample or cohort size. | No | No | Yes | Yes | Yes | No | Yes | Yes | Yes | Yes | Yes |
|  | Define the variables | Yes | Yes | Yes | Yes | Yes | Yes | Yes | Yes | Yes | Yes | No |
|  | Describe the basic statistics of the dataset, particularly of the response variable. | Yes | Yes | Yes | Yes | Yes | Yes | Yes | Yes | Yes | Yes | Yes |
|  | Define how outcomes are measured | Yes | Yes | Yes | Yes | Yes | Yes | Yes | Yes | Yes | Yes | No |
|  | Determine the form of the model (e.g., classification, regression). | Yes | Yes | Yes | Yes | Yes | Yes | Yes | Yes | Yes | Yes | Yes |
|  | Define the success criteria for prediction | No | No | No | No | No | No | Yes | No | Yes | No | No |
|  | Specify the modeling techniques. If only one type of model was used, justify the decision for using that model. | Yes | Yes | Yes | Yes | Yes | Yes | Yes | Yes | Yes | Yes | Yes |
|  | State if outliers with impossible or extreme responses are removed; state any criteria used for outlier removal. | No | No | No | No | No | No | No | No | No | No | No |
|  | State how missing values were handled. | No | No | No | No | Yes | No | Yes | Yes | Yes | Yes | No |
|  | Split the data into a derivation/training set and a validation/ test set. | Yes | Yes | Yes | Yes | Yes | Yes | Yes | Yes | Yes | Yes | Yes |
|  | Specify the model validation strategies | Yes | Yes | Yes | Yes | Yes | Yes | Yes | Yes | Yes | Yes | Yes |
|  | Implement the model selection strategy | Yes | Yes | Yes | Yes | Yes | Yes | Yes | No | Yes | No | No |
|  | External validation should also be performed whenever possible. | No | No | No | No | No | No | No | No | No | No | No |
|  | Report the validation metrics. | Yes | Yes | Yes | Yes | Yes | Yes | Yes | Yes | Yes | Yes | Yes |
| Results | Assess whether sufficient data were available for a good fit of the model | Yes | Yes | Yes | Yes | Yes | Yes | Yes | Yes | Yes | Yes | Yes |
|  | Report the predictive performance of the final model in terms of the validation metrics specified in the methods section. | Yes | Yes | Yes | Yes | Yes | Yes | Yes | Yes | Yes | Yes | Yes |
|  | If possible, report the parameter estimates in the model and their confidence intervals or report non-parametric estimates from bootstrap samples. | No | No | Yes | No | No | No | Yes | No | No | No | Yes |
|  | If possible, report what variables were shown to be predictive/prognostic of the outcome variable. | No | Yes | No | Yes | Yes | Yes | Yes | Yes | Yes | No | Yes |
|  | Report findings from internal/ external validation | Yes | Yes | Yes | Yes | Yes | Yes | Yes | Yes | Yes | Yes | Yes |
| Discus-sion | Interpretation of the final model. | Yes | Yes | Yes | Yes | Yes | Yes | Yes | Yes | Yes | Yes | Yes |
|  | Report the clinical implications derived from the obtained predictive performance. | Yes | Yes | Yes | No | Yes | Yes | Yes | Yes | Yes | Yes | Yes |
|  | Include discussion on (1) balance between model accuracy and model simplicity or interpretability, and (2) the familiarity with the modeling techniques of the end user. | Yes | Yes | No | No | Yes | No | No | Yes | Yes | Yes | No |
|  | Discuss model in context of other models in the literature | Yes | Yes | Yes | Yes | Yes | Yes | No | Yes | Yes | Yes | No |
|  | Potential pitfalls in interpreting the model | Yes | Yes | Yes | No | No | Yes | No | Yes | Yes | Yes | Yes |
|  | Potential bias of the data used in modeling | Yes | Yes | Yes | Yes | Yes | Yes | Yes | Yes | Yes | No | Yes |
|  | Generalizability of the data | Yes | Yes | No | Yes | Yes | No | No | Yes | Yes | Yes | Yes |

| **Section** | **Checklist item** | Mubeen 2017 (19) | Neefjes 2017 (18) | Ng 2012 (36) | Oviedo 2019 (46) | Pei 2019 (17) | Perez-Gandia 2010 (37) | Ramez-ankhani 2017 (16) | Rau 2016 (35) | Scheer 2017 (33) | Toussi 2009 (15) | Zhou 2019 (44) |
| --- | --- | --- | --- | --- | --- | --- | --- | --- | --- | --- | --- | --- |
| Title | Identify the report as introducing a model | No | No | No | Yes | Yes | Yes | Yes | Yes | Yes | No | No |
| Abstract | Background | Yes | Yes | Yes | Yes | Yes | Yes | No | Yes | Yes | Yes | Yes |
|  | Objectives | Yes | Yes | Yes | Yes | Yes | Yes | Yes | Yes | Yes | Yes | Yes |
|  | Data sources | Yes | Yes | Yes | No | Yes | Yes | Yes | Yes | Yes | Yes | Yes |
|  | Machine learning methods applied | Yes | Yes | Yes | No | Yes | Yes | Yes | Yes | Yes | Yes | Yes |
|  | Key result(s) | Yes | Yes | Yes | Yes | Yes | Yes | Yes | Yes | Yes | No | Yes |
|  | Conclusion including the practical value of the model | No | Yes | Yes | Yes | Yes | Yes | No | Yes | Yes | Yes | Yes |
| Intro-duction | Identify the clinical goal | Yes | Yes | Yes | Yes | Yes | Yes | Yes | Yes | Yes | Yes | Yes |
|  | Review the current practice and existing models | Yes | No | Yes | Yes | Yes | Yes | Yes | Yes | Yes | Yes | Yes |
|  | State the nature of study as being a model based on machine learning methods | Yes | Yes | Yes | Yes | Yes | Yes | Yes | Yes | No | Yes | Yes |
|  | Identify how the study may benefit the clinical goal | Yes | Yes | Yes | Yes | Yes | Yes | No | Yes | Yes | Yes | Yes |
| Methods | Identify the clinical setting for the target model. | Yes | Yes | Yes | Yes | Yes | Yes | Yes | Yes | Yes | Yes | Yes |
|  | Specify if the study is retrospective or prospective. | Yes | Yes | Yes | Yes | Yes | No | Yes | Yes | Yes | Yes | Yes |
|  | Specify if the model is prognostic or diagnostic | Yes | Yes | Yes | Yes | Yes | Yes | Yes | Yes | Yes | NA | Yes |
|  | Identify relevant data sources and quote the ethics approval for data access. | No | Yes | Yes | No | Yes | No | Yes | Yes | Yes | Yes | Yes |
|  | State the inclusion and exclusion criteria for data. | Yes | Yes | Yes | No | Yes | No | Yes | Yes | Yes | No | Yes |
|  | Describe the time span of data and the sample or cohort size. | No | Yes | Yes | Yes | Yes | Yes | Yes | Yes | No | Yes | Yes |
|  | Define the variables | Yes | Yes | Yes | Yes | Yes | Yes | Yes | Yes | Yes | Yes | Yes |
|  | Describe the basic statistics of the dataset, particularly of the response variable. | Yes | Yes | Yes | No | Yes | No | Yes | Yes | Yes | Yes | Yes |
|  | Define how outcomes are measured | Yes | Yes | Yes | Yes | Yes | Yes | Yes | Yes | Yes | Yes | Yes |
|  | Determine the form of the model (e.g., classification, regression). | Yes | Yes | Yes | Yes | Yes | Yes | Yes | Yes | Yes | Yes | Yes |
|  | Define the success criteria for prediction | No | No | Yes | Yes | No | No | No | Yes | No | No | No |
|  | Specify the modeling techniques. If only one type of model was used, justify the decision for using that model. | Yes | Yes | Yes | Yes | Yes | Yes | Yes | Yes | Yes | Yes | Yes |
|  | State if outliers with impossible or extreme responses are removed; state any criteria used for outlier removal. | No | No | No | No | No | No | No | No | No | No | No |
|  | State how missing values were handled. | No | No | Yes | Yes | Yes | Yes | Yes | No | Yes | Yes | No |
|  | Split the data into a derivation/training set and a validation/ test set. | Yes | Yes | Yes | Yes | Yes | Yes | Yes | Yes | Yes | Yes | Yes |
|  | Specify the model validation strategies | Yes | Yes | Yes | Yes | No | No | Yes | Yes | Yes | Yes | Yes |
|  | Implement the model selection strategy | Yes | Yes | Yes | Yes | No | No | No | Yes | Yes | No | No |
|  | External validation should also be performed whenever possible. | No | No | No | No | No | No | No | No | No | Yes | No |
|  | Report the validation metrics. | Yes | Yes | Yes | Yes | Yes | Yes | Yes | Yes | Yes | No | Yes |
| Results | Assess whether sufficient data were available for a good fit of the model | No | No | Yes | No | Yes | No | Yes | Yes | Yes | Yes | Yes |
|  | Report the predictive performance of the final model in terms of the validation metrics specified in the methods section. | Yes | Yes | Yes | Yes | Yes | Yes | Yes | Yes | Yes | No | Yes |
|  | If possible, report the parameter estimates in the model and their confidence intervals or report non-parametric estimates from bootstrap samples. | No | Yes | No | No | No | Yes | No | No | No | No | No |
|  | If possible, report what variables were shown to be predictive/prognostic of the outcome variable. | No | Yes | Yes | No | Yes | No | Yes | Yes | Yes | Yes | Yes |
|  | Report findings from internal/ external validation | Yes | Yes | Yes | Yes | No | No | Yes | Yes | Yes | Yes | Yes |
| Discus-sion | Interpretation of the final model. | Yes | Yes | Yes | Yes | Yes | Yes | Yes | Yes | Yes | Yes | Yes |
|  | Report the clinical implications derived from the obtained predictive performance. | No | Yes | Yes | Yes | Yes | Yes | Yes | Yes | Yes | Yes | Yes |
|  | Include discussion on (1) balance between model accuracy and model simplicity or interpretability, and (2) the familiarity with the modeling techniques of the end user. | No | Yes | Yes | No | Yes | No | No | Yes | Yes | No | No |
|  | Discuss model in context of other models in the literature | Yes | Yes | Yes | No | No | No | Yes | Yes | Yes | No | Yes |
|  | Potential pitfalls in interpreting the model | No | Yes | Yes | No | No | Yes | Yes | No | No | No | Yes |
|  | Potential bias of the data used in modeling | No | Yes | Yes | No | Yes | No | Yes | No | Yes | Yes | Yes |
|  | Generalizability of the data | No | No | No | No | No | No | No | No | Yes | No | Yes |
